# The influence of age, gender and socio-economic status on multimorbidity patterns in primary care. first results from the multicare cohort study

**DOI:** 10.1186/1472-6963-12-89

**Published:** 2012-04-03

**Authors:** Ingmar Schäfer, Heike Hansen, Gerhard Schön, Susanne Höfels, Attila Altiner, Anne Dahlhaus, Jochen Gensichen, Steffi Riedel-Heller, Siegfried Weyerer, Wolfgang A Blank, Hans-Helmut König, Olaf von dem Knesebeck, Karl Wegscheider, Martin Scherer, Hendrik van den Bussche, Birgitt Wiese

**Affiliations:** 1 Department of Primary Medical Care, University Medical Center Hamburg-Eppendorf, Martinistr. 52, 20246 Hamburg, Germany; 2 Department of Medical Biometry and Epidemiology, University Medical Center Hamburg-Eppendorf, Martinistr. 52, 20246 Hamburg, Germany; 3 Department of Psychiatry and Psychotherapy, University of Bonn, Sigmund-Freud-Straße 25, 53105 Bonn, Germany; 4 Department of General Practice, Medical Faculty, University of Rostock, 18055 Rostock, Germany; 5 Institute for General Practice, University of Frankfurt am Main, Theodor-Stern-Kai 7, 60590 Frankfurt am Main, Germany; 6 Institute for General Practice, University of Jena, Bachstraße 18, 07743 Jena, Germany; 7 Institute for Social Medicine, Occupational Health and Public Health, University of Leipzig, Semmelweisstr. 10, 04103 Leipzig, Germany; 8 Central Institute of Mental Health, J 5, 68159 Mannheim, Germany; 9 Institute of General Practice, Technical University of Munich, Ismaninger Str. 22, 81675 Munich, Germany; 10 Department of Medical Sociology and Health Economics, University Medical Center Hamburg-Eppendorf, Martinistr. 52, 20246 Hamburg, Germany; 11 Institute for Biometry, Hannover Medical School, 30623 Hannover, Germany

## Abstract

**Background:**

Multimorbidity is a phenomenon with high burden and high prevalence in the elderly. Our previous research has shown that multimorbidity can be divided into the multimorbidity patterns of 1) anxiety, depression, somatoform disorders (ADS) and pain, and 2) cardiovascular and metabolic disorders. However, it is not yet known, how these patterns are influenced by patient characteristics. The objective of this paper is to analyze the association of socio-demographic variables, and especially socio-economic status with multimorbidity in general and with each multimorbidity pattern.

**Methods:**

The MultiCare Cohort Study is a multicentre, prospective, observational cohort study of 3.189 multimorbid patients aged 65+ randomly selected from 158 GP practices. Data were collected in GP interviews and comprehensive patient interviews. Missing values have been imputed by hot deck imputation based on Gower distance in morbidity and other variables. The association of patient characteristics with the number of chronic conditions is analysed by multilevel mixed-effects linear regression analyses.

**Results:**

Multimorbidity in general is associated with age (+0.07 chronic conditions per year), gender (-0.27 conditions for female), education (-0.26 conditions for medium and -0.29 conditions for high level vs. low level) and income (-0.27 conditions per logarithmic unit). The pattern of cardiovascular and metabolic disorders shows comparable associations with a higher coefficient for gender (-1.29 conditions for female), while multimorbidity within the pattern of ADS and pain correlates with gender (+0.79 conditions for female), but not with age or socioeconomic status.

**Conclusions:**

Our study confirms that the morbidity load of multimorbid patients is associated with age, gender and the socioeconomic status of the patients, but there were no effects of living arrangements and marital status. We could also show that the influence of patient characteristics is dependent on the multimorbidity pattern concerned, i.e. there seem to be at least two types of elderly multimorbid patients. First, there are patients with mainly cardiovascular and metabolic disorders, who are more often male, have an older age and a lower socio-economic status. Second, there are patients mainly with ADS and pain-related morbidity, who are more often female and equally distributed across age and socio-economic groups.

**Trial registration:**

ISRCTN89818205

## Background

Over the last decade, a noticeable deal of epidemiological research has concentrated on multimorbidity in the elderly. In most studies multimorbidity means the presence of several chronic diseases in one person for a longer period of time [1]. The main reasons for the growing scientific interest in multimorbidity are the assumption that multimorbidity is a different quality and not just the sum of single diseases [2], the high prevalence and the impact on the affected population, which includes decline of functional status, lower quality of life, higher mortality, increased health care utilization and therefore rising costs of care [3,4].

The prevalence of multimorbidity is dependent on the study design and operationalizations, i.e. the cut-off point (e.g. if multimorbidity is defined by a minimum of 2 or 3 diagnoses), the spectrum of diagnoses included in the studies and the data source (e.g. general population or patients from general practice). Prevalence estimates are highest when using a low cut-off point, an open list of diagnoses and data from general practice [5]. We defined multimorbidity as presence of three or more chronic conditions in order to avoid that almost every patient aged 65 or more is defined as multimorbid [6]. In our previous analysis of insurance claims data in the MultiCare Claims Study we found a prevalence of multimorbidity in the general population in Germany of 62% in the age group 65+ using the 3 disease criterion and a list of 46 diagnosis groups [7].

The central medical professional for the care of elderly patients with multimorbidity is the general practitioner (GP) due to his broad expertise but also to the usually long-standing relationship to older patients. The GP has little help in adjusting care for multiple chronic conditions, because clinical practice guidelines are mostly focused on one disease only. Adhering to current clinical practice guidelines in the treatment of multimorbidity may therefore even have adverse effects [8]. Information about the specific elements and processes in multimorbidity, the interactions and possible synergies of the diseases is urgently needed in order to improve the care of elderly patients with multimorbidity.

Many studies report a higher disease load in females and an increase in the number of chronic conditions with age [9]. In general, morbidity and mortality rates seem to be higher in elderly persons with low economic resources and low educational degree [10,11]. The Survey of Health, Aging and Retirement in Europe (SHARE) showed that older Europeans with a low educational level and wealth experience more cardiovascular disease, lung disease, arthritis, disability and higher mortality rates than their high socioeconomic status counterparts [12]. There also is evidence that multimorbidity is more common and more severe in persons with low socio-economic status [9].

Recent research has tried to untangle the multitude of disease associations in the morbidity spectrum of multimorbid patients by identifying dominant multimorbidity clusters mainly based on causal relations between disorders, e.g. common risk factors [13,14]. Our previous research showed that the associations between chronic conditions can be subsumed in three prevalent multimorbidity patterns if accounting for the fact that multimorbidity patterns share some diagnosis groups, influence each other and overlap in a large part of the population. There were slight gender differences in the composition of the patterns and the prevalence of patterns was highly dependent on gender and age [15]. However, yet there is no evidence how the number of chronic conditions in each pattern is influenced by gender, age and socio-economic status of the patients. The objective of this paper therefore is to analyze the influence of socio-demographic data on the number of diseases per patient in general and for each multimorbidity pattern.

## Methods

### Design

The methods of the study have been described in detail in the published study protocol [16]. In short, the study is designed as a multicentre, prospective, observational cohort study of multimorbid patients from general practice. The study aims to a) investigate the development of multimorbidity patterns over time; b) identify resources and risk factors of the patients that influence the course of these patterns; and c) analyse the somatic, psychological and social consequences of these patterns for the patient's quality of life and functional status [16].

### Recruitment

The patients were recruited from 158 GP practices in 8 study centres distributed across Germany (Bonn, Düsseldorf, Frankfurt/Main, Hamburg, Jena, Leipzig, Mannheim and Munich). In each practice we created a list of patients based on the electronic database of the GP. This list encompassed all patients who were born between 1.7.1923 and 30.6.1943 and consulted the GP at least once within the last completed quarter (i.e. 3 month period). From this list we randomly selected 50 patients with multimorbidity and contacted them for written informed consent. Multimorbidity was defined as coexistence of at least three chronic conditions out of a list of 29 diseases [16].

Patients were excluded from the study if they were no regular patients of the participating practice (i.e. in case of accidental consultation of the GP), if they were unable to participate in interviews (especially blindness and deafness) or if they were not able to speak and read German. Further exclusion criteria were residence in a nursing home, severe illness probably lethal within three months according to the GP, insufficient ability to consent (especially dementia) and participation in other studies at the present time.

**Figure 1:**
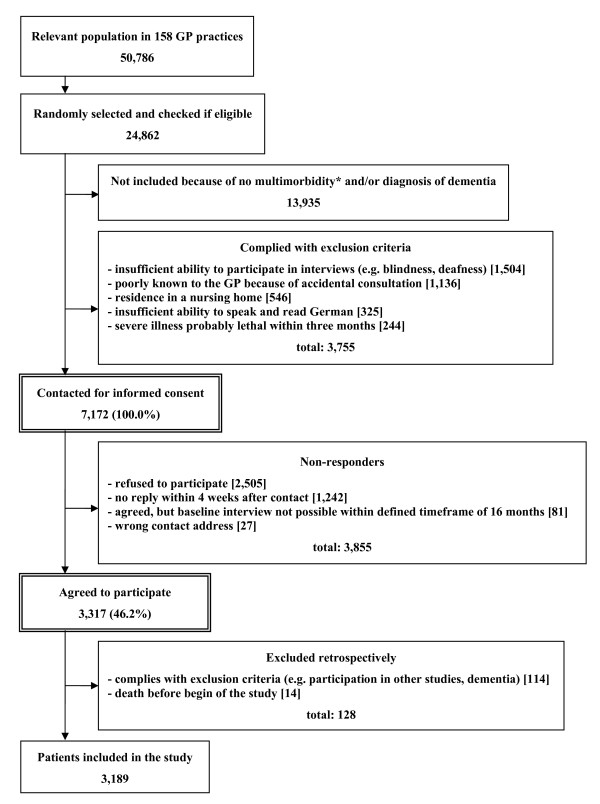
**Sampling and response rate**. * the inclusion criterion "multimorbidity" is defined as at least 3 out of 29 ICD10-based diagnosis groups.

Sampling and response rate of the study are described in Figure 1. Altogether, the relevant population in the participating GP practices comprised of 50,786 patients. A total of 24,862 patients were randomly selected and checked for multimorbidity and exclusion criteria. 13,935 patients were excluded because of no multimorbidity and/or dementia. This equates to 56.0% of the population. Another 3,755 patients were excluded because of the other reasons mentioned above. The remaining 7,172 were contacted for informed consent to participation in our study. From all contacted patients a total of 3,855 did not participate in our study, because they refused to participate, they gave no reply, we could not obtain a valid postal address or they first agreed to participate, but it was not possible to conduct the baseline patient interview within a time frame of 16 months. The other 3,317 patients agreed to participate which corresponds to a total response rate of 46.2%. Retrospectively we had to exclude 128 patients, because they died before the baseline interview or we found out in contact with the patients that they complied with the exclusion criteria without the GP's knowledge. After all, 3,189 patients could be included in the study. Recruitment and baseline data collection took place from July 2008 to October 2009. The study was approved by the Ethics Committee of the Medical Association of Hamburg in February 2008 and amended in November 2008 (Approval-No. 2881).

### Data collection

A comprehensive description of data sources and collected data can be found in the study protocol [16]. For the manuscript in hand we used the patients' morbidity data from the GP, the patients' age and gender from GP charts and socio-demographic data from comprehensive standardized patient interviews, which also included a multitude of other measures described in the study protocol. Additionally we used documentation sheets for basic data of GPs and practices including age, gender and specialty of GP, duration since practice set up, practice type and the number of patients treated in practice.

We conducted a chart review based on ICD10 diagnoses from patients' charts in the electronic documentation system of the GP to determine if the patients were eligible for our study. In anonymous form these data were also used in the non-responder analysis. Morbidity data were assessed using GP interviews regarding the morbidity of study participants using a standardized documentation instrument which covers a list of 46 chronic conditions.

The methods for compiling the list of 46 diagnosis groups have been described elsewhere in detail [7]. In short, we used the most frequent conditions in GP practices as mentioned in a panel survey of the Central Research Institute of Statutory Ambulatory Health Care in Germany ("ADT-Panel") [17]. Chronicity of diagnoses was assessed using the scientific expert report for the formation of a morbidity orientated risk adjustment scheme in the German Statutory Health Insurance [18]. In order to capture a comprehensive picture of the disease patterns in individual patients we amended this list for all chronic conditions with a prevalence ≥ 1% in the age group ≥ 65 years in the data set of the Gmünder ErsatzKasse (GEK) in 2006. The GEK is a German statutory health insurance company with 1.7 million insurants (in 2008), which corresponds to 2.4% of the statutory insured population [19]. For the list of diagnoses ICD-10 codes were grouped together if diseases and syndromes had a close pathophysiological similarity and if ICD codes of related disorders were used ambiguously by coding physicians in clinical reality, respectively. Diagnosis groups and corresponding ICD codes in this list have been published in another paper [15].

The compilation of the list of diagnosis groups was not finished at the beginning of the baseline interviews. For this reason 7 of the 46 diagnosis groups were not part of the standardized GP questionnaire at baseline, but had to be assessed with open questions in the baseline GP interviews ("Which additional diagnoses does that patient have?"). This applies to chronic gastritis, insomnia, allergies, obesity, hypotension, sexual dysfunction, and tobacco abuse. Dementia is not present at baseline, because it served as an exclusion criterion. In subsequent waves of data collection, all 46 diagnosis groups were recorded in standardized form.

The socioeconomic status of the patients (i.e. education, income and former occupation) was assessed with a well-established standardized questionnaire [20]. The highest education grade was described according to the international CASMIN classification in three groups: 1) inadequately completed general education, general elementary education or basic vocational qualification, 2) intermediate qualification or general maturity certificate, and 3) lower or higher tertiary education [21]. The former occupation was grouped according to the degree of autonomy of work [22]. Income was reported as household-size adjusted net income per month, which is calculated as household total net income per month divided by the equivalised household size, which gives 1.0 to the householder, 0.5 to other household members aged 15 or over and 0.3 to each child aged less than 15 years old [20]. The data collection of income was complemented by the assessment of home ownership (i.e. private owned homes in which the patients do not necessarily need to live themselves) serving as a measure of economic advantages or disadvantages accumulated over the life course [23].

### Missing values

We chose a comprehensive data imputation procedure for all variables that will be used in statistical analyses. Missing values in the dataset arising from item non-response have been imputed to avoid bias generated by listwise deletion of subjects with missing values from statistical analyses. We chose the method 'hot deck imputation', which replaces missing values by observed values from a responding unit (donor) that is as similar as possible to the non-responding unit (recipient) regarding characteristics observed in both cases [24]. As donor we chose the nearest neighbour based on Gower distance [25] regarding the auxiliary variables specified below. If there was more than one potential donor with the same distance to the recipient we randomly selected one of these cases. A total of 2,720 patients (85.3%) were eligible as potential donors, i.e. they had complete data sets without any missing values in the auxiliary variables.

As auxiliary variables used for matching donors to recipients we used all items and scores [16] with a proportion of missing values below 2.5%, i.e. gender, age, marital status, household type, education, autonomy at former occupation, home ownership, morbidity measured by 46 diagnosis groups, the four subscales of the Four-Dimensional Symptom Questionnaire [26], the Geriatric Depression Scale [27], the Clinical Dementia Rating Scale [28], the Barthel Index [29], the Instrumental Activities of Daily Living (IADL) scale [30], the motor skills scale FFB-Mot [31], the International Physical Activities Questionnaire [32], the CERAD subscale animal naming [33], the Graded Chronic Pain Scale [34], self-rated health as rated on a 100-point visual analogue scale, health-related quality of life as determined by the EuroQoL EQ-5D [35] using the UK value set [36], body mass index, alcohol use as assessed by AUDIT-C [37], smoking behaviour as measured by pack years, general self-efficacy rated on a standardized scale [38], social support determined by the scale F-SOZU K14 [39] and the quantity of nutrition in relation to the recommendations of German Nutrition Society (DGE) [40].

If auxiliary variables were right-skewed we used their natural logarithm and categorical variables were transformed to dummy variables before matching. After imputation of missing values all variables were refitted to their original scales. We imputed missing values in the following variables: marital status (0.03% missing values), autonomy of work (0.6%), income (12.4%) and home ownership (1.3%). Age, gender, education and household type did not contain any missing values. Imputation of missing values was performed with R 2.13.0 package StatMatch [41].

### Statistical analyses

Descriptive data were presented as means and standard deviations in case of continuous variables and as percentages in case of categorical variables. Diagnosis groups were ranked by prevalence. We excluded missing values from our descriptive analyses and reported the number of available data sets.

Multimorbidity patterns were described according to the results of a factor analysis presented in another paper [15]. In short, these analyses were based on ambulatory data of insurants of the German statutory health insurance company Gmünder ErsatzKasse. Persons were included if they were aged 65 years and older and were permanently insured during the year 2006. The data set used for analyses consisted of 63,104 females and 86,176 males. In the above mentioned paper [15], correlations between diagnosis groups were analyzed by exploratory factor analysis based on a tetrachoric correlation matrix. We used an oblique (oblimin) rotation of factor loading matrices. The factors that result from this analysis can be interpreted as multimorbidity patterns (i.e. clusters of diagnosis groups frequently associated with each other) and each factor loading represents the association of the specific diagnosis group with a pattern. Factors were regarded as substantial if they had an eigenvalue ≥ 1.0. Diagnoses were assigned to a pattern if they had a factor loading of 0.25 or more on the pattern in charge. Additional file 1: Table S1 shows the diagnosis groups assigned to the multimorbidity patterns of both genders including eigenvalues of the factors and factor loadings of diagnosis groups.

For the calculation of prevalences of multimorbidity patterns we assigned individual patients to a pattern if they had diagnoses in at least three groups with a factor loading of 0.25 or more on the corresponding pattern. There were only very few patients with three or more conditions within the pattern of neuropsychiatric disorders, because we had to exclude patients with dementia at baseline. For this reason we excluded the pattern of neuropsychiatric disorders from the figures showing the overlapping of multimorbidity patterns.

We analysed the association of patient characteristics with the number of chronic conditions by multilevel mixed-effects linear regression allowing for random effects at the study centre and GP practice-within-study centre level. Age, gender, marital status, household type, education, degree of autonomy at former occupation, household-size adjusted net income and home ownership were used as independent variables. Before analysis categorical variables were recoded into dummy variables and we made a logarithmic transformation for income, because we supposed a non-linear association for this variable. Separate analyses were conducted for 1) the number of all chronic conditions regardless of the patterns concerned, 2) the number of chronic conditions within the pattern of cardiovascular and metabolic disorders, and 3) within the pattern of anxiety, depression, somatoform disorders (ADS) and pain. Because of the low number of cases within the pattern of neuropsychiatric disorders we did not analyse the morbidity in this pattern separately.

We analyzed whether there were differences between non-responders and study participants using multilevel logistic regression allowing for random effects at the study centre and GP practice-within-study centre level: both groups were compared regarding age, gender and the 29 disease groups used for inclusion. All factors were analyzed in a single multivariate statistical model and were therefore in each case adjusted for the influence of the other variables. Before analysis, age was dichotomized into a) 65 to 74 and b) 75 to 84-years-old patients. For each variable we chose the category with the highest percentage as reference category. The odds ratios resulting from the non-responder analysis were recalculated into risk ratios [42], because risk ratios are easier to interpret. Regarding the chance for enrolment in our study we defined a difference of 25% and more as clinically relevant.

The data used for the non-responder analysis did not contain any missing values. For all other inferential statistics we used complete data sets including imputed data. An α-level of 5% (i.e. p ≤ 0.05) was defined as statistically significant. All statistical tests were conducted using Stata 11.0.

## Results

In total 158 GP practices and 3,189 patients could be included in our baseline assessment. The characteristics of GPs and practices are shown in Table 1. 60.8% of the GPs were male; they had a mean age of 50.2 years and an average of 15.0 years of practice. A total of 67.1% of the GPs had a specialty of family medicine, and 51.3% treated 1.000 or more patients in each quarter (3 months period). 52.5% of the GPs had a single practice. 12.7% conducted practice-sharing (i.e. share their practice with other physicians, but have their own patient base) and 34.8% had a group practice (i.e. share practice and patient base with other GPs).

**Table 1:** Characteristics of GPs and practices (n = 158)

Gender of GP	
- male	60.8%
- female	39.2%
**Age of GP: mean ± sd**	50.2 ± 7.7 years

**Years of practice: mean ± sd**	15.0 ± 8.2 years

**Specialty of GP**	
**- no specialty**	4.4%
**- family medicine ("Facharzt für Allgemeinmedizin")**	65.8%
**- internal medicine ("Facharzt für Innere Medizin")**	28.5%
**- family medicine and internal medicine**	1.3%

**Practice type**	
**- single pactice**	52.5%
**- practice-sharing**	12.7%
**- group practice**	34.8%

**Number of patients treated in practice in each quarter**	
**- 1,000 and more patients**	51.3%
**- 750 thru 999 patients**	24.7%
**- 500 thru 749 patients**	19.6%
**- 499 and less patients**	4.4%

n: number of observations; sd: standard deviation

The socio-demographic data of the study participants are described in Table 2. The patients had a mean age of 74.4 years and 59.3% were female. 62.3% of the study participants had a low degree of education (CASMIN grade 1). The mean household-size adjusted net income per month at the present time was 1412 €, 40.3% were home owners and the average degree of autonomy at their former occupation was 2.9 on a 5 point scale. 56.2% of the patients were married and 57.9% were living with their spouse. 95.6% of the study participants had no nursing dependency and their mean number of chronic conditions was 7.0 out of the list of 46 diagnosis groups.

**Table 2:** Socio-demographic data of patients at baseline (n = 3,189)

Age (at baseline interview): mean ± sd	74.4 ± 5.2 years
**Gender**	
**- male**	40.7%
**- female**	59.3%

**Education (in CASMIN grade)**	
**- grade 1 (low)** **- grade 2 (medium)**	62.3% 26.8%
**- grade 3 (high)**	10.9%

**Household-size adjusted net income per month: mean ± sd**	1412 ± 704 € [n = 2,793]

**Home ownership**	40.3% [n = 3,149]

**Former occupation(in degree of autonomy on a 5 point scale with 1 = low and 5 = high): mean ± sd**	2.9 ± 1.1 [n = 3,128]

**Marital status**	
**- never married**	5.9%
**- married**	56.2%
**- estranged (living in seperate homes)**	2.3%
**- divorced**	8.0%
**- widowed**	27.7% [n = 3,188]

**Household type**	
**- living in private home alone**	35.4%
**- living in private home with spouse**	57.9%
**- living in private home with family members**	4.1%
**- living in private home with other persons**	0.7%
**- living in assisted living**	1.7%
**- living in retirement home**	0.3%
**- living in nursing home**	0**

**Nursing dependency level**	
**- no nursing dependency**	95.6%
**- dependency level 1**	3.4%
**- dependency level 2**	1.0%
**- dependency level 3**	0.1% [n = 3,168]

**Number of chronic conditions** **(based on a list of 46 chronic conditions): mean ± sd**	7.0 ± 2.5

n: number of observations; sd: standard deviation; * exclusion-criterion at baseline

There are slight intercentre differences in the socio-demographic data of the patients. The only outliers are a much lower proportion of home owners in Leipzig (13.4%) and a much higher proportion of medium education (59.4%) against low education (23.0%) in Jena than in the other study centres (cf. Additional file 2 Table S2).

Prevalence and rank order of the diagnosis groups are shown in Table 3. In the total study population, hypertension (prevalence: 77.9%), lipid metabolism disorder (58.5%) and chronic low back pain (49.5%) were the most prevalent diagnoses, which also applies to both age groups and female patients. Male patients also show hypertension and lipid metabolism disorder as most prevalent diagnosis groups, but the third highest prevalence was chronic ischemic heart diseases.

**Table 3:** Prevalence (and rank order) of diagnosis groups by gender and age group

Diagnosis group	total (n = 3,189)	female (n = 1,891)	male (n = 1,298)	age 65-74 (n = 1,827)	age 75-84 (n = 1,362)
Hypertension	77.9% (1)	77.5% (1)	78.4% (1)	74.5% (1)	82.4% (1)

Lipid metabolism disorders	58.5% (2)	57.0% (2)	60.8% (2)	60.5% (2)	56.0% (2)

Chronic low back pain	49.5% (3)	55.2% (3)	41.1% (5)	48.3% (3)	51.0% (3)

Joint arthrosis§	43.3% (4)	48.9% (4)	35.3% (6)	39.4% (4)	48.7% (4)

Diabetes mellitus§	37.6% (5)	33.3% (6)	43.8% (4)	38.8% (5)	36.0% (6)

Thyroid dysfunction§	33.8% (6)	43.5% (5)	19.6% (13)	35.8% (6)	31.1% (7)

Chronic ischemic heart disease§	31.4% (7)	22.2% (12)	44.7% (3)	27.8% (7)	36.1% (5)

Cardiac arrhythmias§	26.9% (8)	23.5% (9)	31.9% (7)	24.0% (9)	30.8% (8)

Asthma/Chronic obstructive pulmonary disease§	24.2% (9)	23.1% (10)	25.7% (9)	24.2% (8)	24.2% (10)

Lower limb varicosis§	23.3% (10)	28.8% (8)	15.2% (17)	21.7% (10)	25.3% (9)

Osteoporosis§	19.8% (11)	28.9% (7)	6.6% (27)	17.5% (13)	23.0% (11)

Severe vision reduction§	18.9% (12)	19.5% (13)	18.2% (14)	16.2% (15)	22.7% (12)

Cancers§	18.3% (13)	15.5% (14)	22.4% (12)	17.6% (12)	19.3% (13)

Depression§	17.8% (14)	22.6% (11)	10.6% (22)	18.6% (11)	16.7% (17)

Purine/pyrimidine metabolism disorders/Gout	17.3% (15)	12.9% (18)	23.7% (10)	16.3% (14)	18.7% (14)

Atherosclerosis/PAOD§	16.7% (16)	12.0% (19)	23.4% (11)	15.2% (16)	18.6% (15)

Intestinal diverticulosis§	14.5% (17)	15.5% (14)	13.0% (20)	14.5% (18)	14.5% (18)

Neuropathies§	14.7% (18)	13.0% (17)	17.3% (15)	15.1% (17)	14.2% (20)

Cardiac insufficiency§	13.1% (19)	11.8% (20)	15.0% (19)	9.6% (22)	17.7% (16)

Chronic gastritis/GERD#	12.9% (20)	13.6% (16)	11.9% (21)	14.0% (19)	11.5% (23)

Cerebral ischemia/Chronic stroke§	11.8% (21)	9.5% (22)	15.1% (18)	9.9% (21)	14.4% (19)

Prostatic hyperplasia	11.4% (22)	-	27.9% (8)	10.5% (20)	12.6% (22)

Renal insufficiency§	10.7% (23)	7.1% (27)	15.8% (16)	8.2% (24)	14.0% (21)

Cardiac valve disorders§	9.4% (24)	8.8% (23)	10.3% (23)	8.0% (26)	11.3% (24)

Chronic cholecystitis/Gallstones	7.9% (25)	8.3% (25)	7.2% (26)	6.7% (28)	9.5% (25)

Dizziness§	7.7% (26)	8.7% (24)	6.3% (29)	6.7% (28)	9.1% (27)

Liver diseases§	7.7% (27)	6.8% (28)	9.0% (25)	8.7% (23)	6.4% (29)

Haemorrhoids	7.5% (28)	5.6% (30)	10.3% (23)	8.1%(25)	6.7% (28)

Urinary incontinence§	7.2% (29)	9.9% (21)	3.3% (36)	5.6% (30)	9.3% (26)

Somatoform disorders§	6.1% (30)	7.7% (26)	3.7% (34)	7.0% (27)	4.9% (32)

Insomnia#	5.6% (31)	5.1% (33)	6.2% (30)	5.4% (32)	5.7% (31)

Severe hearing loss§	5.2% (32)	4.2% (36)	6.8% (28)	4.7% (34)	6.0% (30)

Allergies#	4.9% (33)	6.0% (29)	3.4% (35)	5.8% (29)	3.8% (36)

Obesity#	4.8% (34)	4.8% (35)	4.9% (33)	5.5% (31)	3.9% (35)

Anemias§	4.3% (35)	3.1% (38)	5.9% (31)	3.9% (38)	4.8% (33)

Rheumatoid arthritis/Chronic polyarthritis§	4.2% (36)	5.6% (31)	2.2% (39)	3.9% (37)	4.6% (34)

Anxiety§	4.1% (37)	5.3% (32)	2.2% (39)	4.7% (34)	3.2% (37)

Psoriasis§	3.6% (38)	2.7% (39)	5.0% (32)	4.3% (36)	2.8% (39)

Migraine/chronic headache§	3.5% (39)	4.9% (34)	1.5% (41)	5.0% (33)	1.5% (41)

Noninflammatory gynaecological problems	2.0% (40)	3.4% (37)	-	2.5% (39)	1.4% (42)

Parkinson's disease§	1.9% (41)	1.4% (40)	2.8% (37)	1.3% (41)	2.9% (38)

Urinary tract calculi	1.8% (42)	1.3% (41)	2.6% (38)	1.6% (40)	2.1% (40)

Hypotension#	0.5% (43)	0.5% (42)	0.4% (43)	0.4% (42)	0.6% (43)

Sexual dysfunction#	0.2% (44)	-	0.5% (42)	0.3% (43)	-

Tobacco abuse#	0.1% (45)	0 (43)	0.2% (44)	0.1% (44)	-

Dementias*	-	-	-	-	-

§ used for patient inclusion; # not part of the standardized data collection at baseline, * exclusion-criterion;n: number of cases; GERD: gastroesophageal reflux disease; PAOD: peripheral arterial occlusive disease

### Non-responder analysis

The 3.189 participants in our study were compared with 3.855 non-responders to our study. Patients in the age group 65 to 74 had a 26% higher probability to participate in our study compared to the age group 75 to 84. There were also statistically significant gender differences, but they were below clinical relevance. Regarding morbidity we found statistically significant differences in 8 out of 29 diagnoses used for inclusion, of which 2 diagnoses, namely intestinal diverticulosis (+39%) and psoriasis (+35%) showed a clinically relevant influence on the probability to participate in the MultiCare Cohort Study. For a full description of the results from the non-responder analysis cf. Additional file 3: Table S3.

### Multimorbidity and socio-demographic characteristics

**Figure 2:**
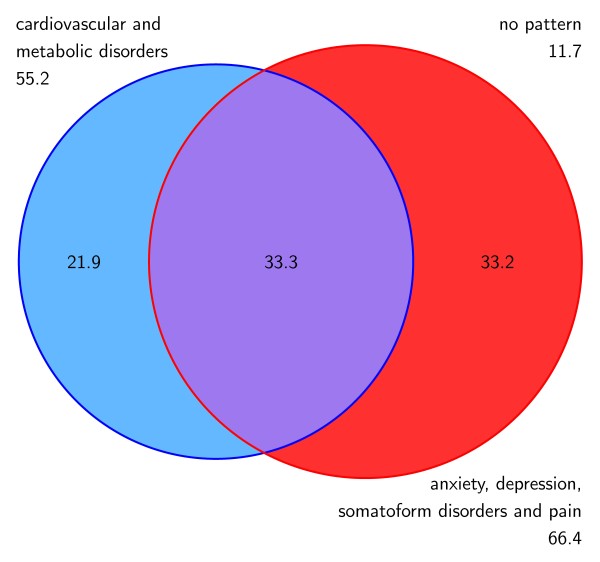
**Overlapping of multimorbidity patterns (in %) related to the female population**.

**Figure 3:**
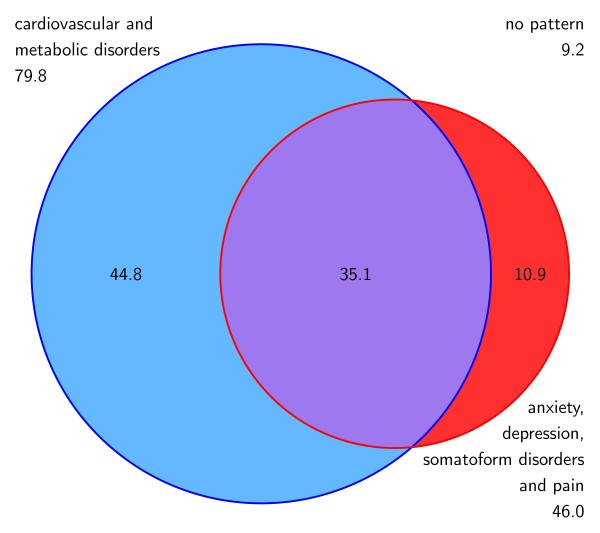
**Overlapping of multimorbidity patterns (in %) related to the male population**.

88.3% of the females and 90.8% of the males with multimorbidity were attributed to at least one of the two multimorbidity patterns. The overlapping of patterns in females and males are shown in Figure 2 and 3. The most prevalent pattern in females is ADS and pain (66.4%), while the most frequent cluster in males is cardiovascular/metabolic disorders (79.8%). Both genders show a comparable proportion of patients that share both patterns (33.3% in females and 35.1% in males, respectively).

The association of the number of chronic conditions and socio-demographic characteristics of the patients is shown in Table 4. The number of chronic conditions in general depends on age (+0.07 conditions per life year over 65), gender (-0.27 conditions for females), education (-0.26 conditions for medium level and -0.29 conditions for high level vs. low level education) and the natural logarithm of income (-0.27 conditions per unit on the logarithmic scale). One step on the logarithmic scale equates to e.g. one of the following steps: from 400 € to 1.100 € to 3,000 € to 8,100 € net income per month. The number of chronic conditions within the pattern of cardiovascular and metabolic disorders shows comparable associations as the number of chronic conditions in general with a higher coefficient for gender (-1.29 conditions for female), while the chronic conditions within the pattern of ADS and pain only correlates with gender (+0.79 conditions for female) and the household type protected institutions (-0.48 conditions for persons living in protected institutions as retirement homes or assisted living vs. persons living at home alone). Marital status did not show any effects on the number of chronic conditions.

**Table 4:** Association of multimorbidity with socio-demographic characteristics: results from multilevel linear regression analyses allowing for random effects at the study centre and GP practice-within-study centre level

	number of all diagnoses		number of diagnoses in CMD		number of diagnoses in ADS/P	
	
	coef (95% CI)	p**	coef (95% CI)	p**	coef (95% CI)	p**
**Age**	***0.07 (0.05/0.08)***	***< 0.001***	***0.04 (0.03/0.06)***	***< 0.001***	0.01 (-0.00/0.02)	0.243

**Gender: female**	***-0.27 (-0.44/-0.09)***	***0.003***	***-1.29 (-1.42/-1.15)***	***< 0.001***	***0.79 (0.66/0.91)***	***< 0.001***

**Marital status (in relation to'never married')**						
**- married**	0.11 (-0.32/0.54)	0.608	0.02 (-0.31/0.35)	0.918	0.09 (-0.23/0.40)	0.590
**- estranged (married, but living in separate homes)**	0.19 (-0.42/0.79)	0.545	-0.13 (-0.59/0.33)	0.582	0.43 (-0.01/0.87)	0.058
**- divorced**	-0.03 (-0.44/0.38)	0.898	-0.02 (-0.33/0.29)	0.910	0.07 (-0.23/0.37)	0.647
**- widowed**	0.12 (-0.23/0.46)	0.511	0.09 (-0.18/0.35)	0.515	0.03 (-0.23/0.28)	0.834

**Household type (in relation to 'living at home alone')**						
**- living at home with spouse**	-0.10 (-0.42/0.23)	0.562	0.05 (-0.20/0.30)	0.678	-0.08 (-0.32/0.16)	0.494
**- living at home with family members or others**	0.24 (-0.14/0.62)	0.210	0.22 (-0.06/0.51)	0.128	-0.07 (-0.34/0.20)	0.611
**- living in assisted living or retirement home**	-0.01 (-0.59/0.57)	0.967	0.27 (-0.17/0.71)	0.231	-***0.48 (-0.90/-0.06)***	***0.024***

**Education (in relation to 'low level')**						
**- medium level**	***-0.26 (-0.45/-0.07)***	***0.008***	***-0.17 (-0.31/-0.02)***	***0.024***	-0.09 (-0.23/0.05)	0.194
**- high level**	-***0.29 (-0.57/-0.01)***	***0.043***	-***0.26 (-0.48/-0.05)***	***0.017***	-0.10 (-0.31/0.10)	0.317

**Degree of autonomy at former occupation (5 point scale)**	-0.02 (-0.10/0.06)	0.571	-0.05 (-0.11/0.01)	0.084	0.04 (-0.02/0.09)	0.221

**Household-size adjusted net income (natural logarithm)**	***-0.27 (-0.47/-0.08)***	***0.005***	***-0.16 (-0.31/-0.01)***	***0.035***	-0.12 (-0.26/0.02)	0.094

**Home ownership**	-0.13 (-0.30/0.05)	0.148	-0.07 (-0.20/0.06)	0.315	-0.03 (-0.15/0.10)	0.658

CMD: cardiovascular and metabolic disorders; ADS/P: anxiety, depression, somatoform disorders and pain; coef: regression coefficient; CI: confidence interval;* statistically significant results (p ≤ 0.05) in italic and bold letters

## Discussion

### Socio-demographic data of multimorbid patients in primary care and their GPs

Our sample consists of 59% females, which is comparable to the proportion of 58% in the general population of Germany aged 65 and more [43]. Marital status and household type seem to reflect the living conditions of the general population in Germany in this age group, e.g. in our study 56% of the patients were married, while in the general population of Western Germany 67% in 65-69 years old to 50% in 75-79 year old persons are married; 58% of the study participants are living together with their spouse, while the proportion in the general population is 73% in 65-69 years old and 53% in 75-59 years old, respectively [44].

The multimorbid patients in our study are mostly treated by experienced GPs. They have a mean practice duration of 15 years, two thirds have a specialty in family medicine and almost a third in internal medicine, only 4% have no specialty. More than half of the practices are large practices with more than 1.000 patients treated per quarter. 35% of GPs in our study have a group practice, which is only slightly below the average proportion in Germany of 39% [45]. The GPs' mean age of 50 years and the gender distribution of 39% females roughly match the average of Germany with 51 years of age and 41% female gender [46].

There is a lower proportion of persons with nursing dependency in our study (4.5%) than can be expected in the general population aged 65+ (5.9% for males and 11.6% for females in 2007) [47]. On the one hand this might be explained by the fact that persons living in nursing homes were excluded in our study. On the other hand an explanation might be that nursing dependency is influenced by age and contrary to general population there are no persons older than 84 years in our study.

Concerning socio-economic status 62% of the patients in our study had low education and only 11% had a high education degree. These education grades seem to be comparable to the elderly general population in GP practices. In AgeCoDe, a study on the early detection of dementia in primary care patients aged 75+ and unselected in regard to morbidity, which had a similar design and similar study centres, there was the same distribution of CASMIN education groups as in the MultiCare Cohort Study [48].

The mean household-size adjusted net income in our study was 1410 €. The Federal Statistics Office of Germany reports a mean net income of 2000 € in men and 1600 € in women aged 70-90 and living alone, and 1500 € if persons in this age group were living with their spouse [49]. In the SHARE study on persons aged 50+ they found an average net income of 1960 € [50], but this study also includes many persons who still have an occupation and therefore should have a higher income than the retired persons in our study. The Berlin Aging Study in patients aged 70+ found a mean net income of 1110 € [51], but the data was limited to Berlin only and they had a clear overrepresentation of higher and highest age groups, which might explain the differences to our study.

Another indicator of social status was wealth measured by home ownership. A proportion of 40% of our study participants were home owners. This is considerably lower than in the SHARE study, where they found that 53% of persons 50+ were home owners [52]. An explanation might be that in contrast to SHARE participants nearly all participants in the MultiCare Cohort Study live in larger cities where the proportion of home owners is lower than in rural areas.

### Morbidity and multimorbidity patterns

In total, 88% of the females and 91% of the males in our study belong to at least one multimorbidity pattern. Females have a lower prevalence of the cardiovascular/metabolic pattern (55% vs. 80% in males) and higher proportion of ADS and pain (66% vs. 46%) than males. In our previous analysis [15] of the general population aged 65 and more we found a much lower prevalence of the patterns than in the multimorbid sample, but the same gender differences, i.e. 48% of females and 51% of males belong to at least one pattern, 30% of females and 39% of males to cardiovascular/metabolic disorder and 34% of females and 22% of males to ADS and pain [15]. The different magnitude of prevalences can be explained by the fact that we included only multimorbid patients in our sample, which represent about 62% in the general population in this age group [7]. The influence of gender that can be presumed from the descriptive analyses is confirmed in the regression analysis, which shows that females in general have 0.3 less diagnoses than males in general and 1.3 diagnoses less in the cardiovascular/metabolic pattern, but 0.8 diagnoses more in the ADS and pain cluster. The lower total number of chronic conditions in female patients is an effect of a multivariate analysis adjusted for the influence of age (and other variables). In a bivariate analysis we found no significant gender differences regarding the total number of diseases.

The gender differences are also reflected in the single diagnosis groups, where most chronic conditions that belong to the cardiovascular/metabolic cluster are less prevalent in the female sample (e.g. chronic ischemic heart diseases: 22% in females vs. 45% in males; atherosclerosis/PAOD: 12% vs. 23%; renal insufficiency: 7% vs. 16%) and diagnoses in the ADS and pain pattern have a higher percentage in the female sample (e.g. thyroid dysfunction: 44% in females vs. 20% in males; lower limb varicosis: 29% vs. 15%; depression: 23% vs. 11%). The only exception with a different direction of gender bias is haemorrhoids, which belongs to the ADS and pain cluster, but is less frequently found in females (6% vs. 10% in males).

The effect of age seems to be relatively small. With each year of age, patients gain 0.07 diagnoses in general and 0.04 diagnoses in the cardiovascular/metabolic cluster, but there was no association of age with the number of diagnoses in the ADS and pain pattern. In accordance with the regression analysis the prevalence of most single diagnoses of the cardiovascular/metabolic pattern slightly increases with age (e.g. cardiac arrhythmias: 24% in the age 65-74 vs. 31% in the age 75-84; cardiac insufficiency: 10% vs. 18%; renal insufficiency: 8% vs. 14%). Important exceptions are diabetes mellitus and purine/pyrimidine metabolism disorders/gout, which both belong to the cardiovascular/metabolic cluster, but have lower prevalence in the higher age groups. Especially regarding diabetes mellitus this might be an effect of selective survival as our analyses at the present time are based on cross-sectional data. In the ADS and pain cluster we did not find consistent results regarding an age-dependent change in prevalence.

There seems to be no effect of marital status and little effect of the household type on the number of chronic conditions. Persons living in protected institutions had a mean of 0.5 diagnoses less in the ADS and pain pattern. This might be explained by underreporting as the care of patients in protected institutions is primarily performed by nurses and the GP has lower communication intensity with this patient group than with patients who live independently. Protected institutions were defined as assisted living and retirement homes, but no nursing homes, as persons in nursing homes had to be excluded at baseline.

Regarding the indicators of socioeconomic status we found that multimorbidity was associated with education and income. Patients with medium education or high education had a mean of 0.3 diagnoses less than persons with low education. This influence was also significant in the cardiovascular/metabolic pattern, where medium education led to 0.2 and high education to 0.3 diagnoses less than low education. Household-size adjusted net income also had an effect on the number of chronic conditions in general (0.3 diagnoses less for each step on the logarithmic scale, e.g. 1.100 to 3.000 Euros per month) and in the cardiovascular/metabolic pattern (0.2 diagnoses less). Former occupation and home ownership showed in bivariate analyses a comparably small effect on the number of chronic condition that disappeared after controlling for the other indicators of socio-economic status. Neither of the variables of socioeconomic status had any effect on the number of diagnoses in the ADS and pain cluster.

### Comparison with other studies

We found a higher number of chronic conditions with increasing age. This is in line with other studies, which found a higher occurrence rate of multimorbidity and a higher number of chronic conditions in the elderly [5,53,54]. But the difference we found was comparatively small. It takes approximately 14 life years over 65 to explain a difference of one chronic condition. Our previous analyses in the MultiCare Claims Study suggest a similarly small effect of age on the number of chronic conditions. In this study the range of 17 life years explained a difference of one chronic condition [7]. However, the small size of the difference in age might also be an effect of survival bias in our cross-sectional analyses, as a higher number of chronic conditions seems to be associated with increased mortality [9]. The relationship between age and the number of chronic conditions will be further investigated as soon as longitudinal data are available.

Our descriptive data analysis suggests that there might be no gender differences in the occurrence of multimorbidity. In multivariate regression analyses we found a higher total number of chronic conditions in male patients. This is contrary to many studies which found that multimorbidity may be more common in females than in males and that female gender seems to be associated with a higher number of chronic conditions [53,55], although the gender difference in some cases was very small [7,56].

The different results in our study might be explained by three factors. First, age seems to be an important confounder in gender differences. Results from bivariate analyses might suggest that females have a higher number of chronic conditions while in fact this is only an effect of the higher mean age of females.

Second, we conducted comprehensive GP interviews, while many other studies rely on patient self-reports or insurance claims data. It has been described that there might be a higher prevalence of multimorbidity amongst females in the general population, while there are more males with multimorbidity in general practice [5].

Third, the gender differences might be an effect of the spectrum of diagnoses included in the studies. Our analyses suggest that gender differences depend on the multimorbidity pattern, i.e. in the ADS and pain cluster females have more diagnoses than males, while in cardiovascular/metabolic pattern females have less diagnoses than males. This also has been reported in our previous analyses [15]. It has been suggested in other studies that the higher frequency of ADS and pain diagnoses in women might reflect the real prevalences of these disorders and not just be a gender-based reporting/detection problem in primary care. [57]

We found no effect of marital status on the health status of our multimorbid cohort. This is contrary to many other studies which showed that married adults may have lower morbidity and better physical health than their unmarried counterparts [58]. There also was no difference in the morbidity load between persons living alone and persons living together with their spouse, which has been suggested by other studies [59]. It might be that both variables only play a minor role for the morbidity load of multimorbid patients.

Regarding socio-economic status we found that income and education, influenced the number of chronic conditions in our study participants. Education also significantly influenced occurrence and extent of multimorbidity in a number of other studies [53,60,61]. Regarding the influence of (former) education we found an association in a bivariate analysis that disappeared in a multivariate analysis, probably because of a correlation with the education degree. Other studies found inconsistent results, either no significant results regarding occupation-based socio-economic status [60] or higher occurrence of multimorbidity in some occupation-based groups [62]. In an overall view, our findings are in line with other studies, indicating that low socio-economic status is associated with a higher number of chronic conditions [9,54].

### Strengths and weaknesses

The MultiCare Cohort Study is focused on elderly multimorbid patients from general practice. We decided to include only patients with at least three chronic conditions, which is a higher illness burden than required in most other studies. The reason for this decision was that we wanted to avoid that almost every patient in the age group 65+ was defined as multimorbid. The data from our sampling procedure shows that - despite this restriction - our definition of multimorbidity still applies to 44% of the patients in this age group. For this reason our results are clinically relevant as the data represent a large number of patients from general practice.

We were able to obtain a high participation rate of 46%. In the similarly designed AgeCoDe study on patients aged 75+, but unselected with regard to morbidity, they obtained a participation rate of 50% [48]. The slightly lower response rate in our study might have occurred because of a higher morbidity burden in our patients.

We performed a non-responder analysis in order to investigate the selection bias in our study. There is a higher percentage of younger patients than in general practice, i.e. patients aged 65 to 74 have a 26% higher chance for participation in our study than patients aged 75 to 84. We also found a statistically significant and clinically relevant higher proportion of patients with intestinal diverticulosis (with a 39% higher chance) and psoriasis (with a 35% higher chance) although we have no definite medical explanation for these differences. In spite of these results, we have no selection bias in 27 of 29 diagnosis groups, which include chronic conditions with high illness burden as cancer, stroke and depression, and there is also no gender bias in the recruitment of study participants.

Factors that may affect the generalizability of the MultiCare Cohort Study may result from our exclusion criteria. We had to exclude patients with dementia at baseline, because of their inability to consent. For this reason the extent of the neuropsychiatric pattern is underestimated and we had to exclude this pattern from our baseline analyses. We also had to exclude patients residing in a nursing home. Finally, we recruited patients only in larger German cities, so that rural areas are not covered from our study.

The results in this paper are based on baseline data only, i.e. at the present time we only performed cross-sectional data analyses. Thus, it was not possible to decide which causal direction lies behind the statistical associations we found. Additionally, as already mentioned above, some differences (and also: missing differences) between age groups might - at least in part - be an effect of selective survival. To investigate bias from cross-sectional design we will replicate our analyses with longitudinal data as soon as data from follow-up waves are available.

A strength of our study relates to a high data quality that results from the fact that interviewers were regularly trained and monitored and a multitude of procedures for prevention of insufficient data quality, detection of inaccurate or incomplete data and actions to improve data quality were performed, e.g. user reliability trainings, automatic plausibility and integrity checks and data error reports to the collaborating centres.

The morbidity data were assessed in GP practices, which have been shown to be a less biased data source than patient self-reports [9]. But it also has been shown that the validity of diagnoses from German GP charts may be impaired by both, underreporting and overreporting. Underreporting mainly related to symptoms and less severe diagnoses frequently encountered in GP practice. Overreporting mostly applied to suspected, but not clinically confirmed diagnoses of chronic conditions [63]. The reliability of GP self-documentation (i.e. postal interviews) in cohort studies seems to be rather low. Over the course of 4.5 years, 19% of the diagnoses of diabetes mellitus, 35% of coronary heart disease, and 45% of stroke disappeared in the GP documentation of the AgeCoDe Study [64]. To obtain a good data quality regarding morbidity, we decided to combine both data sources - GP charts and GP interviews, use a standardized questionnaire as reminder and conduct the interviews face-to-face. The reliability and validity of this approach will be assessed and published when data from the first follow-up are available.

Another strength of our approach relates to a comprehensive picture of chronic diseases in the individual patients. We included all highly prevalent chronic conditions (≥ 1% in the age group 65+) into our 46 diagnosis groups. For that reason we are quite sure that our statistical model is adjusted for noticeable influences of confounding diagnoses that may bias our results.

Additional strengths consist of multivariate analyses dealing with possible confounding, multilevel models allowing for cluster effects and an advanced treatment of missing values. We used recruitment by chart registry not waiting-room recruitment, therefore we should have no problems with overestimation of conditions that lead to greater heath care utilization.

## Conclusions

We found that female gender is not generally associated with a higher morbidity load as many studies suggest - instead it seems to depend on the type of multimorbidity considered. Women seem to be more vulnerable to ADS and pain-related morbidity while men might more often suffer from cardiovascular and metabolic diseases. As reported in other studies, we found that older age is associated with a greater number of chronic conditions, but this effect seems to be rather small. Low socio-economic status might also lead to a greater extent of multimorbidity. Its best predictors are education and income, which both are independently associated with multimorbidity. The effect of age and deprivation seems to be limited to a part of the morbidity spectrum. Both, older age and low socio-economic status were associated with a higher number of cardiovascular/metabolic disorders, but we found no influence of these variables on the number of diagnoses in the ADS and pain pattern. Contrary to other studies we found no effect of living arrangements and marital status on the morbidity load of our population.

In summary, there seem to be at least two types of elderly multimorbid patients. First, there are patients with mainly cardiovascular and metabolic disorders, who are more often male, have an older age and a lower socio-economic status. Second, there are patients mainly with ADS and pain-related morbidity, who are more often female, but equally distributed across age and socio-economic groups.

Future analyses will show if the development of these patterns is influenced by different resources and risk factors (e.g. nutrition, social support or self efficacy) and which somatic, psychological and social consequences these cluster have. This evidence might help us to facilitate diagnosis, amend prevention, lower costs in health care systems and increase the quality of life in elderly multimorbid patients.

## Competing interests

The authors declare that they have no competing interests.

## Authors' contributions

HvdB, IS, HH, KW, MS and BW conceived and designed the study. BW and GS prepared the data for analysis. IS and GS analysed the data. IS drafted the manuscript. SH, AA, AD, JG, SRH, SW, WB, HHK, and OvdK participated in study design and implementation. All authors read and approved the final manuscript.

## Pre-publication history

The pre-publication history for this paper can be accessed here:

http://www.biomedcentral.com/1472-6963/12/89/prepub

## Supplementary Material

Additional file 1 **Table S1**. Multimorbidity patterns by gender* - results from tetrachoric factor analyses. Click here for file

Additional file 2 **Table S2**. Intercentre differences in socio-demographic data of patients at baseline (n = 3,189). Click here for file

Additional file 3 **Table S3**. Comparison of study participants and non-responders regarding the chance for study participation: results from multilevel logistic regression analysis allowing for random effects at the study centre and GP practice-within-study centre level. Click here for file
