# Supplementary material for: The influence of age, gender and socio-economic status on multimorbidity patterns in primary care. first results from the multicare cohort study
Source: BMC Health Serv Res. 2012 Apr 3;12:89. doi: 10.1186/1472-6963-12-89 (PMC3348059; doi:10.1186/1472-6963-12-89)
Supplement: Additional file 1 — Table S1. Multimorbidity patterns by gender* - results from tetrachoric factor analyses. [file 1472-6963-12-89-S1.DOC]

Additional file 1: Multimorbidity patterns by gender* – results from tetrachoric factor analyses§

|  | **Females** | **Males** |
| --- | --- | --- |
| **Cardiovascular and metabolic disorders** | Eigenvalue: 6,34  Associated diagnosis groups (factor loadings):   - Hypertension (,71) - Purine/pyrimidine metabolism disorders /Gout (,60) - Diabetes mellitus (,55) - Obesity (,52) - Renal insufficiency (,47) - Lipid metabolism disorders (,45) - Liver diseases (,39) - Chronic ischemic heart diseases (,38) - Atherosclerosis/PAOD (,35) - Cardiac valve disorders (,31) - Chronic cholecystitis/Gallstones (,29) - Cardiac arrhythmias (,28) - *Urinary tract calculi (,28)* - Cardiac insufficiency (,26) | Eigenvalue: 1,96  Associated diagnosis groups (factor loadings):   - Hypertension (,70) - Diabetes mellitus (,59) - Renal insufficiency (,55) - Chronic ischemic heart diseases (,53) - Purine/pyrimidine metabolism disorders /Gout (,50) - Atherosclerosis/PAOD (,50) - Lipid metabolism disorders (,49) - Obesity (,47) - Cardiac insufficiency (,46) - Liver diseases (,40) - Cardiac valve disorders (,37) - Cardiac arrhythmias (,37) - *Tobacco abuse (,32)* - *Cerebral ischemia/Chronic stroke (,30)* - *Anemias (,30)* - *Neuropathies (,27)* - Chronic cholecystitis/Gallstones (,25) |
| **Anxiety, depression, somatoform disorders and pain** | Eigenvalue: 2,37  Associated diagnosis groups (factor loadings):   - Chronic low back pain (,61) - Hypotension (,58) - Migraine/Chronic headache (,53) - *Noninflammatory gynecological  problems (,52)* - Somatoform disorders (,51) - Hemorrhoids (,45) - Depression (,44) - Anxiety (,39) - Allergies (,38) - Osteoporosis (,37) - Chronic gastritis/GERD (,36) - Joint arthrosis (,35) - Intestinal diverticulosis (,32) - Insomnia (,31) - Lower limb varicosis (,31) - Dizziness (,30) - *Rheumatoid arthritis /Chronic Polyarthritis (,29)* - Thyroid dysfunction (,27) - Asthma/COPD (,27) | Eigenvalue: 7,14  Associated diagnosis groups (factor loadings):   - Chronic low back pain (,66) - Hypotension (,54) - Somatoform disorders (,58) - Hemorrhoids (,51) - Migraine/Chronic headache (,49) - Depression (,47) - Anxiety (,47) - Joint arthrosis (,44) - *Prostatic hyperplasia (,43)* - Allergies (,41) - Intestinal diverticulosis (,40) - Chronic gastritis/GERD (,40) - Insomnia (,37) - Dizziness (,37) - *Sexual dysfunction (,35)* - Osteoporosis (,35) - Lower limb varicosis (,33) - *Urinary tract calculi (,30)* - Thyroid dysfunction (,27) - Asthma/COPD (,27) - *Severe hearing loss (,27)* |
| **Neuropsychiatric disorders** | Eigenvalue: 1,71  Associated diagnosis groups (factor loadings):   - Dementias (,78) - Parkinson's disease (,56) - Cardiac insufficiency (,51) - Urinary incontinence (,49) - Cerebral ischemia/Chronic stroke (,43) - Depression (,36) - *Renal insufficiency (,33)* - *Chronic ischemic heart diseases (,31)* - *Dizziness (,30)* - *Anemias (,29)* - *Atherosclerosis/PAOD (,25)* | Eigenvalue: 1,75  Associated diagnosis groups (factor loadings):   - Dementias (,70) - Parkinson's disease (,60) - Urinary incontinence (,55) - Cerebral ischemia/Chronic stroke (,38) - Depression (,33) - Cardiac insufficiency (,31) |

*** Gender-specific associations of diagnosis groups with a pattern are presented in italic letters.**§ **Factor loadings < .25 have been omitted. PAOD: peripheral arterial occlusive disease;
COPD: chronic obstructive pulmonary disease; GERD: gastroesophageal reflux disease**
