# Supplementary material for: The influence of age, gender and socio-economic status on multimorbidity patterns in primary care. first results from the multicare cohort study
Source: BMC Health Serv Res. 2012 Apr 3;12:89. doi: 10.1186/1472-6963-12-89 (PMC3348059; doi:10.1186/1472-6963-12-89)
Supplement: Additional file 2 — Table S2. Intercentre differences in socio-demographic data of patients at baseline (n = 3,189). [file 1472-6963-12-89-S2.DOC]

Additional file 2: Intercentre differences in socio-demographic data of patients at baseline (n=3,189)

|  | **Bonn**  **(n=422)** | **Dusseldorf**  **(n=399)** | **Frankfurt/Main**  **(n=269)** | **Hamburg**  **(n=452)** | **Jena**  **(n=244)** | **Leipzig**  **(n=488)** | **Mannheim**  **(n=493)** | **Munich**  **(n=422)** |
| --- | --- | --- | --- | --- | --- | --- | --- | --- |
| **Age in years (at baseline interview): mean ± sd** | 74.3 ± 5.0 | 74.4 ± 5.0 | 75.1 ± 5.3 | 74.6 ± 5.2 | 74.2 ± 5.2 | 74.0 ± 5.1 | 73.9 ± 5.3 | 74.9 ± 5.4 |
| **Gender: female** | 58.8% | 61.2% | 59.9% | 60.2% | 60.7% | 59.6% | 56.2% | 59.2% |
| **Education (in CASMIN grade)**  **- grade 1 (low)**  **- grade 2 (medium)**  **- grade 3 (high)** | 63.0%  24.2%  12.8% | 74.4%  19.3%  6.3% | 66.9%  27.9%  5.2% | 61.5%  28.8%  9.7% | 23.0%  59.4%  17.6% | 58.6%  20.9%  20.5% | 74.4%  18.9%  6.7% | 60.7%  31.3%  8.1% |
| **Household size adjusted net income in €: mean ± sd** | 1582 ± 731 | 1422 ± 861 | 1528 ± 820 | 1450 ± 708 | 1115 ± 448 | 1249 ± 368 | 1380 ± 561 | 1538 ± 883 |
| **Home ownership** | 56.7% | 36.4% | 48.3% | 40.9% | 48.9% | 13.4% | 45.7% | 42.5% |
| **Former occupation (1=low / 5=high): mean ± sd** | 3.0 ± 1.1 | 2.8 ± 1.2 | 2.9 ± 1.1 | 3.2 ± 1.0 | 2.8 ± 1.1 | 3.1 ± 1.1 | 2.7 ± 1.2 | 2.7 ± 1.1 |
| **Marital status**  **- never married**  **- married**  **- estranged (living in seperate homes)**  **- divorced**  **- widowed** | 5.2%  58.9%  1.9%  8.3%  25.7% | 3.3%  53.4%  2.5%  9.3%  31.6% | 7.1%  49.8%  1.5%  7.4%  34.2% | 6.4%  57.3%  1.8%  7.7%  26.8% | 4.9%  63.9%  1.6%  4.5%  25.0% | 5.1%  60.3%  1.8%  8.4%  24.4% | 5.3%  57.6%  3.5%  7.1%  26.6% | 10.0%  48.1%  2.8%  9.7%  29.4% |
| **Household type**  **- living in private home alone**  **- living in private home with spouse**  **- living in private home with family members**  **- living in private home with other persons**  **- living in assisted living**  **- living in retirement home** | 32.9%  60.4%  2.6%  0.2%  3.8%  0 | 39.6%  56.4%  3.0%  0.3%  0.8%  0 | 40.9%  49.8%  6.7%  0.4%  1.1%  1.1% | 36.1%  56.2%  3.1%  1.1%  2.7%  0.9% | 23.4%  65.2%  10.7%  0  0.8%  0 | 33.2%  62.1%  2.5%  0.6%  1.6%  0 | 32.9%  61.1%  3.7%  1.2%  1.2%  0 | 41.9%  51.2%  5.0%  1.0%  0.7%  0.2% |
| **Nursing dependency level**  **- no nursing dependency**  **- dependency level 1**  **- dependency level 2**  **- dependency level 3** | 93.4%  5.5%  1.2%  0 | 96.2%  3.8%  0  0 | 94.6%  3.5%  1.9%  0 | 96.0%  2.7%  1.3%  0 | 95.7%  3.4%  0.4%  0.4% | 97.3%  2.1%  0.6%  0 | 94.7%  3.5%  1.6%  0.2% | 96.0%  3.1%  0.7%  0.2% |
| **Number of chronic conditions*: mean ± sd** | 6.9 ± 2.4 | 7.3 ± 2.7 | 6.6 ± 2.1 | 7.0 ± 2.3 | 6.5 ± 2.2 | 7.2 ± 2.5 | 7.1 ± 2.4 | 7.3 ± 2.7 |
