# Supplementary material for: The influence of age, gender and socio-economic status on multimorbidity patterns in primary care. first results from the multicare cohort study
Source: BMC Health Serv Res. 2012 Apr 3;12:89. doi: 10.1186/1472-6963-12-89 (PMC3348059; doi:10.1186/1472-6963-12-89)
Supplement: Additional file 3 — Table S3. Comparison of study participants and non-responders regarding the chance for study participation: results from multilevel logistic regression analysis allowing for random effects at the study centre and GP practice-within-study centre level. [file 1472-6963-12-89-S3.DOC]

Additional file 3: Comparison of study participants and non-responders regarding the chance for study participation: results from multilevel logistic regression analysis allowing for random effects at the study centre and GP practice-within-study centre level

|  | **Participants  (n=3,189)** | **Non-responders  (n=3,855)** | **Risk ratio**  **[95% CI]** | **p*** |
| --- | --- | --- | --- | --- |
| Gender: male | 40.7% | 35.1%  [n=3,850] | 1,16 [1,09-1,24] | < 0.001 |
| ***Age < 75 when contacted for informed consent*** | ***55.5%*** | ***44.3%***  ***[n=3,526]*** | ***1,26 [1,21-1,31]*** | ***< 0.001*** |
| Diagnosis groups used for patient inclusion |  |  |  |  |
| - Joint arthrosis | 38.1% | 35.7% | 1,13 [1,06-1,20] | < 0.001 |
| - Diabetes mellitus | 36.9% | 40.1% | 0,92 [0,86-0,99] | 0.017 |
| - Thyroid dysfunction | 32.4% | 31.5% | 1,09 [1,01-1,17] | 0.031 |
| - Chronic ischemic heart disease | 30.9% | 30.3% | 1,03 [0,95-1,11] | 0.475 |
| - Cardiac arrhythmias | 26.2% | 24.3% | 1,10 [1,00-1,19] | 0.039 |
| - Asthma/COPD | 24.3% | 23.4% | 1,05 [0,96-1,15] | 0.261 |
| - Cancers | 21.8% | 19.6% | 1,10 [0,99-1,21] | 0.065 |
| - Lower limb varicosis | 21.7% | 22.7% | 0,95 [0,86-1,05] | 0.338 |
| - Osteoporosis | 19.0% | 17.9% | 1,17 [1,05-1,30] | 0.005 |
| - Atherosclerosis/PAOD | 17.7% | 16.4% | 1,01 [0,90-1,13] | 0.882 |
| - Severe vision reduction | 16.4% | 15.9% | 1,08 [0,96-1,21] | 0.213 |
| - Depression | 15.3% | 17.4% | 0,93 [0,83-1,05] | 0.238 |
| - Neuropathies | 14.8% | 13.8% | 1,13 [1,00-1,28] | 0.055 |
| - (Alcoholic) liver diseases and alcohol abuse | 13.1% | 14.4% | 0,83 [0,72-0,95] | 0.006 |
| ***- Intestinal diverticulosis*** | ***12.3%*** | ***8.5%*** | ***1,39 [1,20-1,60]*** | ***< 0.001*** |
| - Cardiac insufficiency | 10.6% | 11.5% | 0,96 [0,83-1,11] | 0.611 |
| - Cerebral ischemia/Chronic stroke | 10.4% | 9.7% | 1,07 [0,92-1,25] | 0.362 |
| - Renal insufficiency | 9.6% | 9.1% | 1,05 [0,89-1,23] | 0.554 |
| - Cardiac valve disorders | 8.8% | 7.2% | 1,18 [0,99-1,39] | 0.064 |
| - Urinary incontinence | 6.0% | 6.0% | 1,14 [0,93-1,39] | 0.196 |
| - Somatoform disorders | 5.9% | 6.3% | 0,90 [0,73-1,10] | 0.292 |
| - Dizziness | 5.6% | 5.7% | 1,03 [0,83-1,26] | 0.802 |
| - Severe hearing loss | 5.1% | 5.0% | 0,99 [0,79-1,23] | 0.902 |
| - Anemias | 4.9% | 4.9% | 1,04 [0,83-1,29] | 0.762 |
| - Anxiety | 4.1% | 4.4% | 0,89 [0,69-1,13] | 0.337 |
| - Migraine | 3.7% | 3.8% | 0,98 [0,76-1,27] | 0.905 |
| ***- Psoriasis*** | ***3.7%*** | ***2.7%*** | ***1,35 [1,03-1,77]*** | ***0.031*** |
| - Rheumatoid arthritis/Chronic polyarthritis | 3.5% | 3.6% | 1,00 [0,77-1,30] | 0.984 |
| - Parkinson’s disease | 1.9% | 1.4% | 1,40 [0,95-2,05] | 0.088 |

**n: number of observations; 95% CI: 95% confidence interval; * statistically significant (p ≤ 0.05) and clinically relevant results (RR ≤ 0.75 and ≥ 1.25, respectively) in italic and bold letters.**
